# Catastrophic costs and impoverishment among tuberculosis-affected households in Nepal: results from a national patient cost survey

**DOI:** 10.1093/heapol/czag095

**Published:** 2026-07-24

**Authors:** Prajowl Shrestha, Naveen Prakash Shah, Sharad Kumar Sharma, Lok Raj Joshi, Ashish Lal Shrestha, Padmanav Ghimire, Basundhara Sharma, Rajendra Basnet, Ratna Bahadur Bhattarai, Sandesh Neupane, Rabin Gautam, Prasant Vikram Shahi, Barsha Thapa, Kenza Bennani, Bir Bahadur Rawal, Keshab Deuba

**Affiliations:** National Tuberculosis Control Center, Ministry of Health and Food Safety, Thimi, Bhaktapur, Nepal; National Tuberculosis Control Center, Ministry of Health and Food Safety, Thimi, Bhaktapur, Nepal; National Tuberculosis Control Center, Ministry of Health and Food Safety, Thimi, Bhaktapur, Nepal; International Union Against Tuberculosis and Lung Disease (The Union), Kathmandu, Nepal; Save the Children, Kathmandu, Nepal; National Tuberculosis Control Center, Ministry of Health and Food Safety, Thimi, Bhaktapur, Nepal; National Tuberculosis Control Center, Ministry of Health and Food Safety, Thimi, Bhaktapur, Nepal; Save the Children, Kathmandu, Nepal; Save the Children, Kathmandu, Nepal; Public Health Professional, Kathmandu, Nepal; World Health Organization (WHO), Kathmandu, Nepal; World Health Organization (WHO), Kathmandu, Nepal; World Health Organization (WHO), Kathmandu, Nepal; World Health Organization (WHO), Kathmandu, Nepal; National Tuberculosis Control Center, Ministry of Health and Food Safety, Thimi, Bhaktapur, Nepal; Public Health and Environment Research Center (PERC), Lalitpur, Nepal; Department of Global Public Health, Karolinska Institutet, Stockholm, Sweden; Global Health Epidemiology Group, Centre for International Health (CIH), Department of Global Public Health and Primary Care, University of Bergen, Bergen, Norway; Bergen Centre for Ethics and Priority Setting in Health (BCEPS), Department of Global Public Health and Primary Care, University of Bergen, Bergen, Norway

**Keywords:** Nepal, tuberculosis, catastrophic costs, impoverishment, patient cost survey, social protection

## Abstract

Tuberculosis (TB) poses a significant economic burden on affected households. Despite the provision of free diagnosis and treatment services, patients and their households experience substantial financial hardship. This study aimed to estimate the catastrophic costs faced by TB patients in Nepal and identify associated factors. We conducted the first national cross-sectional survey of 1000 patients enrolled within the National Tuberculosis Program (NTP). The sample consisted of 600 drug-susceptible (DS-TB) and 400 drug-resistant TB (DR-TB) patients. Data were collected through structured interviews using the standardized TB patient cost survey questionnaire. Costs were categorized as direct medical, direct nonmedical, and indirect. Indirect costs were estimated using the human-capital approach. Catastrophic total costs due to TB were defined as total TB-related costs exceeding 20% of annual pre-TB household income. The mean age of participants was 43 years, and 65% were male. Forty-two percent of participants engaged in informal paid work and 70% had not completed secondary education. One-third of the participants reported having some form of social protection, with just 11% possessing health insurance coverage. Overall, 51% of TB-affected households faced catastrophic costs due to TB, with a higher proportion among DR-TB patients (76%) compared with DS-TB patients (51%). The financial burden was primarily driven by direct nonmedical costs (44%) and indirect costs (39%), substantially exceeding direct medical costs (17%). Factors associated with catastrophic total costs included belonging to a poorer wealth quintile, a DR-TB diagnosis, being the primary income earner, and lacking health insurance coverage. TB-related costs pushed 13% of households below the poverty line, increasing to 31% among DR-TB households. Mitigating this burden requires an integrated approach that aligns early diagnostic interventions with comprehensive social protection. Specifically, combining direct cash transfers for wage replacement with in-kind nutritional support is critical to protect high-risk households and reduce nonmedical financial shocks.

Key Messages

- Tuberculosis (TB) continues to impose substantial financial hardship on households in Nepal despite free diagnosis and treatment, with more than half of TB-affected households experiencing catastrophic costs.
- Nonmedical expenses and income loss are the dominant components of this financial burden, indicating that financial protection gaps exist primarily outside the health service delivery system.
- TB-related costs pushed 13% of households below the poverty line, increasing to 31% among drug-resistant TB households, indicating that TB care must be integrated with poverty-sensitive social protection.
- Strengthening social protection and patient support, including targeted cash and nutritional assistance alongside clinical TB care, could help Nepal progress toward the end TB target of zero catastrophic costs.

## Introduction

Despite being a preventable and curable disease, tuberculosis (TB) remains a major global public health priority, with an estimated 10.7 million people falling ill with the disease in 2024 (WHO 2025a), and it disproportionately affects populations living in poverty (World Health Organization 2005). During the same year, TB was responsible for ∼1.23 million deaths globally. The burden is particularly acute in the WHO South-East Asia Region, which accounted for ∼34% of global incident TB cases in 2024.

The burden is heavily concentrated in resource-constrained settings, with the 30 high TB burden countries accounting for 87% of all estimated cases globally in 2024 (WHO 2025a). TB disproportionately affects individuals in low- and middle-income countries, where social determinants such as malnutrition, overcrowded living conditions, and limited access to healthcare are prevalent (Lönnroth et al. 2009, Raviglione and Krech 2011, Siroka et al. 2016, Duarte et al. 2018).

In Nepal, the TB epidemic remains a substantial challenge. According to the latest estimates, ∼67 000 people developed TB in 2024, representing an incidence rate of 227 per 100 000 population (WHO 2025a). Furthermore, TB caused an estimated 16 000 deaths in the country in 2024. Nepal is listed among the 30 high burden countries for multidrug-resistant or rifampicin-resistant TB (MDR/RR-TB; WHO 2025a).

Healthcare financing in Nepal operates through a mixed system reliant on government revenues, external development assistance, and substantial out-of-pocket payments by households. Although free basic healthcare services and the national health insurance program aim to improve access and financial protection, out-of-pocket spending continues to account for a large share of current health expenditure (59.4%; World Bank 2025). This limited financial protection may leave socioeconomically vulnerable households exposed to catastrophic costs when seeking care for prolonged and complex illnesses, such as TB.

The Government of Nepal has adopted the End TB Strategy and set a target of ensuring that no TB-affected families incur catastrophic costs by 2035. In recent years, Nepal has introduced innovative, people-centered approaches and endorsed the “TB Free Initiative” which aims to strengthen local government commitment and accountability in accelerating efforts to end TB (National Tuberculosis Control Centre 2021). Nepal has made progress to increase access to healthcare and reduce the financial burden on people through the implementation of social protection measures such as free packages of basic healthcare and national health insurance. The National Tuberculosis Program (NTP) provides economic support to patients with drug-resistant TB (DR-TB), including assistance for hostel accommodation, nutrition, and travel.

While the link between low socioeconomic status and catastrophic health expenditure is well-recognized globally, national-level evidence in Nepal has been lacking. Previous subnational research provided limited insights (Gurung et al. 2021, Bengey et al. 2023) but did not offer the comprehensive data required to inform federal policy. This study represents the first nationally representative survey in Nepal to quantify the social and economic impact of TB on affected households. By establishing a national baseline, this research provides evidence to monitor Nepal’s progress toward the WHO End TB Strategy target of zero catastrophic costs for TB-affected households. Furthermore, it identifies factors associated with financial hardship, which remain high despite the provision of free diagnosis and treatment. These findings are instrumental for the NTP to design targeted social protection mechanisms and prioritize resources under the “TB Free Initiative” to protect the most vulnerable populations from further impoverishment.

## Materials and methods

### Study design and population

We conducted a facility-based, cross-sectional survey to assess catastrophic costs and impoverishment experienced by TB patients and their households. The study population comprised TB patients of all age groups who were enrolled in and receiving treatment through the NTP. Eligible participants included patients with any TB treatment history who had received at least 14 days of anti-TB therapy at the time of interview. To ensure national representation, the survey was conducted across three distinct ecological zones: the Mountain region, the Hill region, and the Terai (plains) region. Data collection occurred at the facility level.

### Sampling design and sample size

We employed a cluster sampling design, with health facilities providing TB services serving as the primary sampling units. A total of 1000 participants were recruited, comprising 600 DS-TB patients and 400 DR-TB patients. Sample size calculations and cluster selections were conducted in accordance with the guidelines and calculation tools provided in the WHO handbook for Tuberculosis Patient Cost Surveys (WHO 2017).

For the DS-TB cohort, clusters were selected from a sampling frame of 2833 eligible primary sampling units using a probability-proportional-to-size (PPS) method based on 2021/2022 notification data. A total of 50 clusters were selected. These 50 clusters included 106 health facilities, as some health facilities were combined into a single cluster based on patient volume. The sample size was calculated based on a total notified cases population of 37 700 patients, an estimated 60% prevalence of catastrophic costs, a design effect of 2, and an absolute precision of 6%. This calculation yielded a minimum required sample of 509 patients. To account for potential nonresponse and incomplete data, a 10% allowance for nonresponse and incomplete data was applied, increasing the target to 560. To maintain equal representation, 12 patients were randomly selected within each of the 50 clusters from the sampling frame developed using patient client codes recorded in the electronic TB register. This resulted in a final sample of 600 DS-TB patients.

For the DR-TB cohort, the target population was substantially smaller, with 662 notified cases nationally in 2021/2022. Due to the limited number of treatment centers managing drug-resistant cases, a PPS method was not utilized. Instead, a comprehensive inclusion approach was applied, selecting all 20 facilities that had recorded DR-TB patients during the reference period. The sample size projection assumed an 80% prevalence of catastrophic costs, a design effect of 2, and an absolute precision of 5%. This calculation determined a minimum required sample size of 359 patients. Incorporating a 10% allowance for nonresponse and incomplete data adjusted the required target to 395. We randomly selected 20 eligible patients from each of the 20 clusters using patient codes recorded in the DHIS2 Tracker system. This resulted in a final sample of 400 DR-TB patients.

### Data collection tools and process

Data collection was conducted using a structured interview approach based on the generic instrument provided in the WHO’s TB Patient Cost Survey handbook. Twenty trained field researchers administered the structured interviews between 20 January and 28 March 2024. The reporting of this study was guided by the consolidated health economic evaluation reporting standards (CHEERS) 2022 checklist. The standardized WHO questionnaire was adopted and structurally retained, covering all recommended domains: sociodemographic and clinical data extracted from treatment cards; out-of-pocket expenditures and time lost prior to diagnosis; expenditure and time lost during the current treatment phase; and household coping mechanisms and social protection access.

To ensure contextual validity, the generic instrument was adapted for use in Nepal. Specific adaptations included the translation of the tool into Nepali, the contextualization of healthcare delivery tiers, and the adjustment of financial variables to the local currency (Nepalese Rupee). Additional questions regarding social protection were tailored to capture specific local schemes.

Prior to main survey implementation, the questionnaire was pretested on the digital Kobo Toolbox platform using the KoboCollect application. A 6-day training program was conducted to orient and train the field team on the study protocol, interview techniques, tools, ethical considerations, and the use of the digital data collection system. The average duration of each interview was ∼60–90 min. A monitoring checklist was used to conduct on-site monitoring to ensure data quality and adherence to the study protocol.

Consistent with the cross-sectional design, data were collected through a single interview conducted at various points during the treatment course. The study sample included participants who were in either the intensive phase or the continuation phase of their DS-TB or DR-TB regimens at the time of the survey. To minimize recall bias, pretreatment costs, which include all expenses incurred from the onset of symptoms until the day of treatment initiation, were only collected from patients interviewed during the intensive phase.

Specific interview protocols were implemented based on the age and economic role of the participants. For minors, interviews were conducted with their parents or legal guardians, who served as proxy respondents. For adult participants who were not the primary breadwinners of their household, patients remained the primary respondents for questions concerning their individual diagnostic pathway, personal time loss, and direct out-of-pocket expenditures. To guarantee the accuracy of the broader economic metrics, such as total household income and household-level coping mechanisms, these participants were encouraged to consult with the primary breadwinner or head of household when completing the financial sections of the questionnaire.

### Estimation of catastrophic total costs due to tuberculosis

Catastrophic total costs due to TB were estimated using the WHO End TB Strategy indicator. The numerator included direct medical costs, direct nonmedical costs, and indirect costs incurred during the TB episode. The denominator was annual pre-TB household income. The self-reported monthly pre-TB household income was annualized by multiplying by 12. Four missing or implausible monthly household-income values were replaced with the mean monthly household income of respondents in the same asset-based wealth quintile before annualization. Catastrophic total costs due to TB were defined as total TB-related costs exceeding 20% of annual pre-TB household income.

Direct medical costs included out-of-pocket payments for consultations, laboratory tests, and medications. Direct nonmedical costs comprised expenditures for transportation, food, accommodation, and nutritional supplements or additional food. Indirect costs represented the economic value of time lost due to illness and care-seeking and were estimated using the human-capital approach.

Total costs for the entire TB episode were estimated by aggregating three components: pretreatment costs, intensive-phase costs, and continuation-phase costs. Because patients were interviewed once rather than followed longitudinally, costs for unobserved or incomplete treatment phases were extrapolated. Phase-specific median monthly costs were calculated separately by treatment phase and TB type, and multiplied by the planned duration of the corresponding treatment phase.

Patient time loss was valued using self-reported pre-TB monthly individual income divided by reported working hours. Where working hours were missing or zero but income was reported, a 160-h working month was assumed. For unemployed participants reporting zero pre-TB individual income, patient time loss was valued at a nominal hourly value of NPR 1. This conservative human-capital boundary was used to avoid simulating income losses to participants with no pre-TB earnings, while retaining caregiver wage losses and patient time losses among participants with pre-TB earnings in the indirect cost estimate. This assumption affected only the valuation of the patient’s own time loss and was not used for caregiver time valuation or for the household-income denominator. For participants employed in the informal sector, productivity loss was calculated using self-reported average monthly earnings before the onset of TB. Caregiver time, where reported, was valued using Nepal’s monthly minimum wage of NPR 17 300 divided by 160 working hours, equivalent to NPR 108 per hour. Thus, the national minimum wage was applied only to caregiver time valuation.

### Impoverishment and wealth stratification

Impoverishment was assessed by estimating the poverty headcount before and after TB-related costs. We used the updated World Bank international extreme poverty line of US$3 per person per day in 2021 purchasing power parity (PPP; Alfani et al. 2025). For Nepal, this was converted to Nepali Rupees using the World Bank World Development Indicators private-consumption PPP conversion factor for 2021. The household-level poverty threshold was calculated by multiplying the annual per-person threshold by household size. Households were classified as poor before TB-related costs if their annual pre-TB household income was below this threshold, and as poor after TB-related costs if annual pre-TB household income minus total TB-related costs was below the same threshold.

Wealth quintiles were constructed from household asset ownership and dwelling characteristics using principal component analysis. The resulting asset index was divided into five quintiles and used to examine socioeconomic gradients in impoverishment and other equity-stratified outcomes.

### Data management and analysis

To ensure the completeness of data, mandatory responses, validation rules and skip patterns were implemented on the Kobo Toolbox platform. We analyzed the data using Stata/BE 17.0, following the WHO patient cost survey handbook to ensure adherence to standardized methodologies (WHO 2017).

To ensure the national representativeness of the results, we applied weights that accounted for the multistage cluster sampling design and the unequal probability of selection between the DS-TB and DR-TB cohorts. The application of these weights allowed for the calculation of point estimates that reflect the national burden of TB-related costs.

We described the study population using descriptive statistics and calculated mean and median out-of-pocket direct medical, direct nonmedical costs, and indirect costs before TB treatment initiation and during TB treatment. For continuous data, median, mean, interquartile range (IQR), and 95% confidence interval (95% CI) were presented where appropriate, and frequency distribution tables were produced for categorical data. Multivariable logistic regression models were used to assess catastrophic costs due to TB. Models were specified using a directed acyclic graph (DAG)-informed approach, with separate exposure-specific models for primary income earner status, insurance status, and TB type. Covariates were selected based on their role as potential confounders.

### Ethical considerations

Written informed consent was obtained from all adult participants prior to the interview. For participants under 18 years of age, written informed consent was obtained from their parents or legal guardians, and age-appropriate assent was obtained from the participants. Their confidentiality and privacy were also maintained throughout the data management and analysis processes.

## Results

### Sociodemographic characteristics

A total of 1000 TB (600 DS-TB and 400 DR-TB) patients participated in the study. The overall mean age of participants was 42.7 years. The largest age groups were 15–24 years (19%) and 65 years or older (18%). Children younger than 15 years accounted for 5.4% of the total participants. Around 65% of the total participants were men, and the most common occupational category was informal paid work (42%). About 70% of participants had not completed secondary school education. The median household size was 5, with similar distributions in the DS-TB and DR-TB groups (Table 1).

**Table 1:** Sociodemographic characteristics of TB patients in Nepal, 2024 (*N* = 1000).

Characteristics	All participants *N* = 1000 (%)	DS-TB participants *n* = 600 (%)	DR-TB participants *n* = 400 (%)
Gender			
Female	352 (35.4)	223 (37.2)	129 (32.8)
Male	648 (64.6)	377 (62.8)	271 (67.2)
Age in years, mean (95% CI)	42.7 (40.8–44.6)	42.2 (39.8–44.6)	43.4 (40.3–46.4)
Age category, years			
0–14	54 (5.4)	50 (8.3)	4 (1.0)
15–24	191 (19.1)	109 (18.2)	82 (20.5)
25–34	151 (14.6)	79 (13.2)	72 (16.7)
35–44	140 (13.9)	74 (12.3)	66 (16.2)
45–54	140 (14.4)	84 (14.0)	56 (15.0)
55–64	147 (14.1)	91 (15.2)	56 (12.7)
65+	177 (18.5)	113 (18.8)	64 (17.9)
Education level			
No education or illiterate	250 (25.8)	152 (25.3)	98 (26.4)
Literate but no formal education	108 (10.8)	63 (10.5)	45 (11.1)
Primary/lower secondary school	292 (29.1)	156 (26.0)	136 (33.7)
Secondary/higher secondary and above	308 (30.3)	198 (33.0)	110 (26.6)
Not yet started school	42 (4.0)	31 (5.2)	11 (2.2)
Occupation (pre-TB)			
Unemployed	173 (17.3)	117 (19.5)	56 (14.0)
Formal paid work	59 (5.9)	35 (5.8)	24 (6.0)
Informal paid work	420 (42.0)	221 (36.8)	199 (49.6)
Retired	13 (1.3)	9 (1.5)	4 (1.0)
Student	74 (7.4)	46 (7.7)	28 (7.1)
Housework	184 (18.4)	120 (20.0)	64 (16.1)
Other: foreign employment	21 (2.1)	10 (1.7)	11 (2.8)
Other: self-employed business	54 (5.4)	40 (6.7)	14 (3.4)
Other	2 (0.2)	2 (0.3)	0 (0)
Household size, median (95% CI)	5 (4.8–5.2)	5 (4.8–5.2)	5 (4.6–5.4)

Counts are unweighted; percentages, means, medians, and 95% CIs are weighted estimates. DS-TB and DR-TB are domain estimates.

### Socioeconomic characteristics

One-third (36%) of the participants were the primary income earner in their households. The median pre-TB monthly household income was NPR 28 000 for DS-TB households and NPR 27 000 for DR-TB households (Table 2). The asset-based wealth distribution suggested that DR-TB participants were relatively more socioeconomically disadvantaged than DS-TB participants. Regarding social protection, including social security schemes, health insurance or allowances, 32% of all participants (21% among DS-TB and 50% among DR-TB) were enrolled in a social protection scheme and only 11% reported having health insurance.

**Table 2:** Socioeconomic characteristics of TB patients and their households in Nepal, 2024 (*N* = 1000).

Socioeconomic characteristics	All participants *N* = 1000 (%)	DS-TB participants *n* = 600 (%)	DR-TB participants *n* = 400 (%)
Primary income earner			
Yes	356 (35.6)	197 (32.8)	159 (39.8)
No	644 (64.4)	403 (67.2)	241 (60.2)
Wealth quintile			
Quintile 1 (least poor)	200 (20.0)	127 (21.2)	73 (18.3)
Quintile 2	198 (19.8)	138 (23.0)	60 (15.0)
Quintile 3	213 (21.3)	115 (19.2)	98 (24.4)
Quintile 4	192 (19.2)	119 (19.8)	73 (18.3)
Quintile 5 (poorest)	197 (19.7)	101 (16.8)	96 (24.0)
Social protection			
Yes^a^	324 (32.4)	124 (20.7)	200 (50.0)
No	676 (67.6)	476 (79.3)	200 (50.0)
Insurance status			
Insured^b^	112 (11.2)	61 (10.2)	51 (12.7)
Not insured	888 (88.8)	539 (89.8)	349 (87.3)
**(Pre-TB) Personal income** ^c^, median (95% CI)	Rs 5000 (3329–6671)	Rs 5000 (3260–6740)	Rs 8000 (4889–11 111)
**(Pre-TB) Household income**, median (95% CI)	Rs 27 000 (25 747–28 253)	Rs 28 000 (25 564–30 436)	Rs 27 000 (24 926–29 074)
**(Pre-TB) Household income**, median (95% CI)			
Quintile 1 (least poor)	Rs 50 000 (43 573–56 427)	Rs 50 000 (43 186–56 814)	Rs 50 000 (36 751–63 249)
Quintile 2	Rs 30 000 (26 841–33 159)	Rs 30 000 (26 643–33 358)	Rs 35 000 (28 456–41 544)
Quintile 3	Rs 27 000 (24 188–29 812)	Rs 25 000 (21 533–28 467)	Rs 27 000 (21 345–32 655)
Quintile 4	Rs 21 000 (18 852–23 148)	Rs 20 000 (17 702–22 298)	Rs 25 000 (21 078–28 923)
Quintile 5 (poorest)	Rs 16 000 (14 459–17 541)	Rs 16 000 (13 634–18 366)	Rs 16 000 (12 737–19 263)

Counts are unweighted; percentages, means, medians, and 95% CIs are weighted estimates. DS-TB and DR-TB are domain estimates.

^a^Social protection refers to being enrolled in any social security, health insurance, or allowance.

^b^Insurance status refers to being enrolled in any public or private health insurance.

^c^Those who were unemployed and had zero income were also included.

### Clinical characteristics

Overall, 79% of the total participants were new cases (Table 3). The proportion of new cases among DS-TB participants was 93% and among DR-TB was 58%. Approximately 71% of the DS-TB participants had pulmonary TB. More than half of the DS-TB (68%) and DR-TB (52%) participants were in the continuation phase of their treatment. Regarding treatment modality, self-administered treatment was utilized by 85% of the total participants, with 93% of DS-TB patients and 73% of DR-TB patients following this approach. Nearly 48% of DR-TB participants were receiving the longer treatment regimen. Extensively drug-resistant tuberculosis accounted for 0.8% of DR-TB participants. Overall, 26.4% of participants had TB with comorbidities, with 28% among DS-TB and 24% among DR-TB participants.

**Table 3:** Clinical characteristics of TB patients in Nepal, 2024 (*N* = 1000).

Clinical characteristics	All participants *N* = 1000 (%)	DS-TB participants *n* = 600 (%)	DR-TB participants *n* = 400 (%)
TB treatment phase			
Intensive phase	370 (37.7)	187 (31.2)	183 (47.6)
Continuation phase	630 (62.3)	413 (68.8)	217 (52.4)
Treatment status			
New case	796 (78.9)	559 (93.2)	237 (57.6)
Previously treated patients	204 (21.1)	41 (6.8)	163 (42.4)
Treatment modality			
Self-administration^a^	836 (84.9)	556 (92.7)	280 (73.4)
DOTS	164 (15.1)	44 (7.3)	120 (26.6)
DS-TB type (*n* = 600)			
Pulmonary bacteriologically confirmed	–	344 (57.4)	–
Pulmonary clinically diagnosed		83 (13.8)	
Extra-pulmonary		173 (28.8)	
DR-TB type—treatment regimen (*n* = 400)			
MDR/RR (SSTR)	–	–	135 (30.6)
MDR/RR (LTR)			179 (48.0)
Pre-XDR			83 (20.6)
XDR			3 (0.8)
Comorbidity^b^			
Without comorbidity	731 (73.6)	431 (71.8)	300 (76.3)
With one disease	237 (23.2)	148 (24.7)	89 (21.1)
With two diseases	32 (3.2)	21 (3.5)	11 (2.6)

Counts are unweighted; percentages are weighted estimates.

DOTS, directly observed treatment, short-course; MDR/RR (LTR), multidrug-resistant or rifampicin-resistant TB treated with a Longer Treatment Regimen; MDR/RR (SSTR), multidrug-resistant or rifampicin-resistant TB treated with a Shorter Standardized Treatment Regimens; XDR, extensively drug-resistant TB.

^a^Self-administration is defined as a process by which a person with TB takes TB medicine from a DOTS facility, without supervision from health workers but under the supervision of family members.

^b^Co-morbidity includes HIV, diabetes, chronic obstructive pulmonary disease, cancer, and chronic kidney disease.

### Costs of tuberculosis-affected households

#### Costs incurred throughout the treatment course

The median direct medical cost for all participants was NPR 6377 (IQR: 5000–14 857), with DR-TB participants incurring a median medical cost of NPR 6837 and DS-TB patients incurring NPR 6377 (Table 4). The median direct nonmedical cost was NPR 44 281 (IQR: 36 506–70 364) for all participants, with DR-TB participants incurring NPR 73 915 (IQR: 44 857–122 157), and DS-TB participants incurring NPR 39 183 (IQR: 32 600–47 954). Furthermore, the median indirect cost for people with DS-TB was NPR 3384 (IQR: 486–33 212) and for people with DR-TB was NPR 24 856 (IQR: 4821–73 987).

**Table 4:** TB-related costs incurred by TB-affected households over the treatment course in Nepal, 2024 (*N* = 1000).

	All participants		DS-TB participants		DR-TB participants	
	Mean (95% CI)	Median (IQR)	Mean (95% CI)	Median (IQR)	Mean (95% CI)	Median (IQR)
Direct medical cost	16 173 (11 748–20 598)	6377 (5000–14 857)	15 834 (11 315–20 352)	6377 (6100–11 213)	30 678 (20 622–40 733)	6837 (4810–24 834)
Direct nonmedical cost	42 129 (38 955–45 304)	44 281 (36 506–70 364)	40 471 (37 403–43 539)	39 183 (32 600–47 954)	113 008 (87 229–138 786)	73 915 (44 857–122 157)
Indirect cost	37 847 (25 926–49 769)	12 107 (844–46 820)	37 204 (25 015–49 392)	3384 (486–33 212)	65 369 (47 428–83 309)	24 856 (4821–73 987)
Total cost	96 150 (82 125–110 174)	78 397 (47 557–149 582)	93 509 (79 262–107 756)	60 845 (44 045–97 227)	209 054 (165 283–252 825)	130 974 (73 558–209 067)

Costs are presented in Nepali rupees (NPR). Means, medians, IQRs, and 95% CIs are weighted estimates.

**Figure 1:**
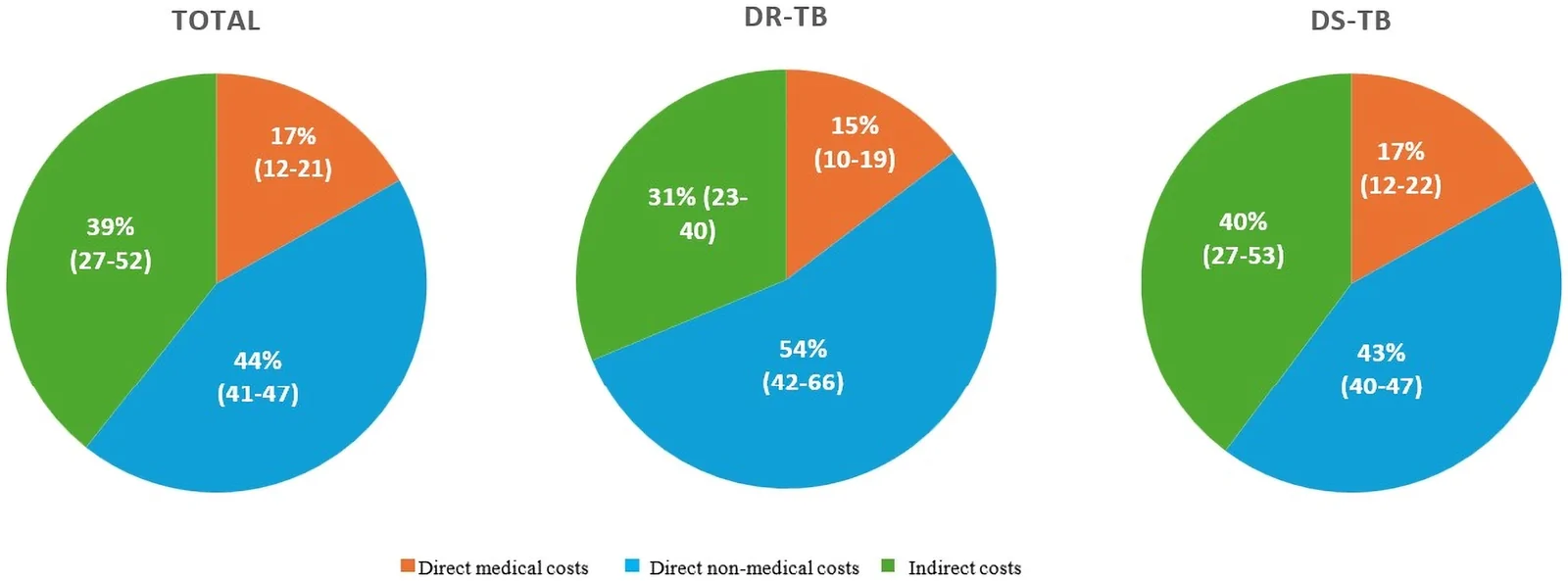
Proportion of cost distribution among TB-affected households in Nepal, 2024 (*N* = 1000).

**Figure 2:**
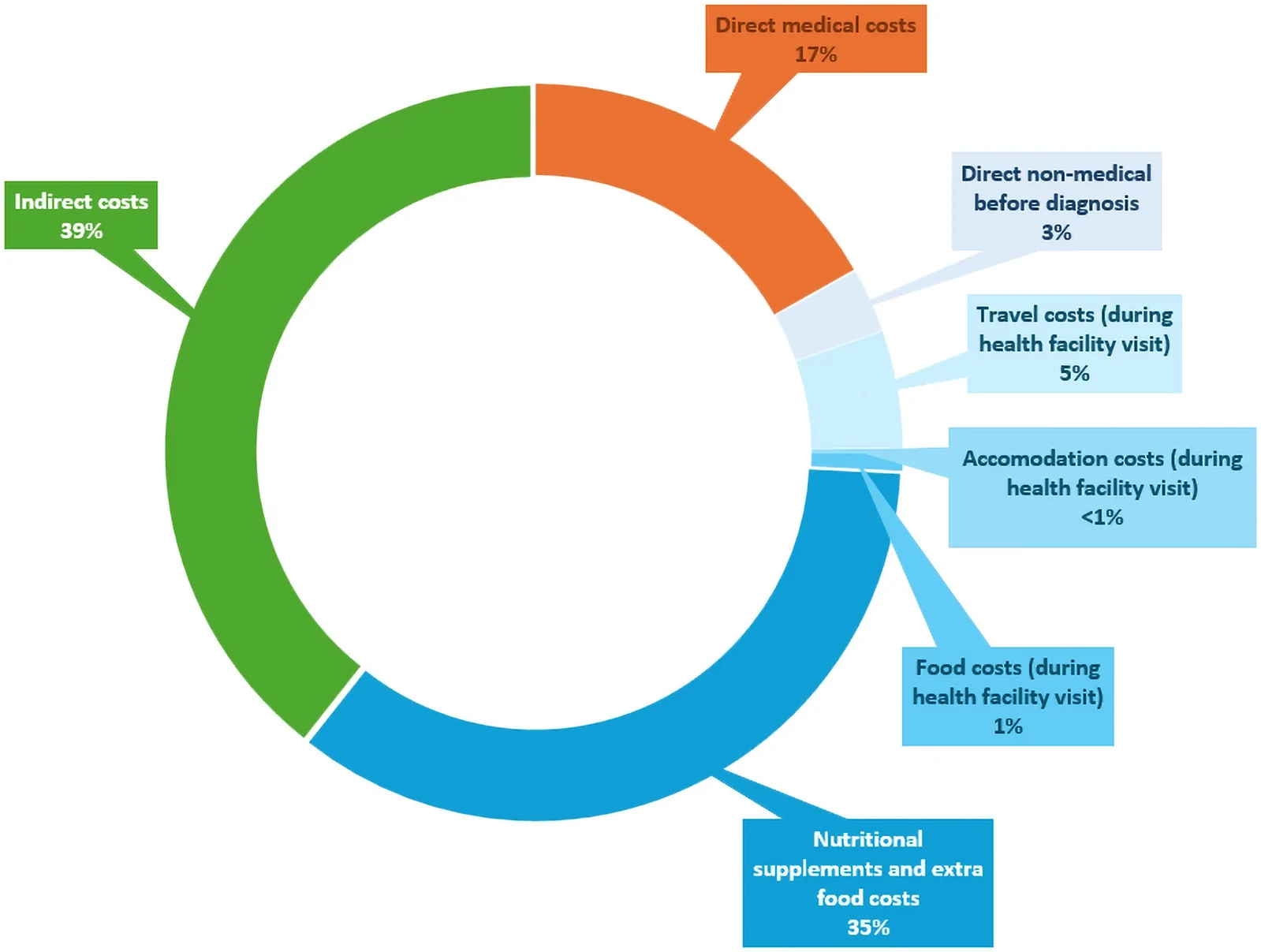
Distribution of total costs by cost category among TB-affected households in Nepal, 2024 (*N* = 1000).

The largest cost driver was direct nonmedical costs, accounting for 44% of total expenses among all participants, 54% among DR-TB patients, and 43% among those with DS-TB (Figs 1 and 2).

This was followed by indirect costs, or lost income, making up 39% of total costs, with 31% for DR-TB and 40% for DS-TB. Of the total TB-related costs, 35% was attributed to nutritional supplements and additional food consumed during the TB episode. Supplementary Table S1 describes the distribution of nutritional supplement and extra food expenditure. Supplementary Figure S1 presents weighted mean costs by category and TB type. The weighted mean expenditure on nutritional supplements and extra food was NPR 33 496 overall, NPR 33 119 among DS-TB patients, and NPR 49 601 among DR-TB patients (Supplementary Table S1 and Supplementary Figure S1). The weighted median was NPR 35 506 across groups, while the upper tail was higher among DR-TB patients, with a 95th percentile of NPR 112 580 compared with NPR 61 486 among DS-TB patients. Weighted zero expenditure was 3.1% overall, 3% among DS-TB patients and 9.1% among DR-TB patients.

#### Cost distribution in different treatment phases

The median direct medical costs among total participants during the prediagnosis phase were NPR 6100 and during the postdiagnosis intensive and postdiagnosis continuation phases were NPR 1086 and NPR 466, respectively (Table 5). The median cost of nutritional supplements and extra food intake following diagnosis was NPR 20 784 in intensive phase and NPR 35 506 in continuation phase. Similarly, the median cost for lost income in the prediagnosis phase was NPR 500, in postdiagnosis intensive phase was NPR 2946 and in postdiagnosis continuation phase was NPR 2583.

**Table 5:** Detailed breakdown of costs incurred by TB patients across the prediagnosis and treatment phases in Nepal, 2024 (*N* = 1000).

	All participants *N* = 1000		DS-TB participants *n* = 600		DR-TB participants *n* = 400	
	Mean (95% CI)	Median (IQR)	Mean (95% CI)	Median (IQR)	Mean (95% CI)	Median (IQR)
Prediagnosis						
Direct medical	10 541 (8492–12 589)	6100 (4810–6100)	10 562 (8466–12 657)	6100 (6100–6100)	9649 (7505–11 793)	4810 (4810–4810)
Travel	931 (756–1106)	500 (500–750)	919 (741–1097)	500 (500–500)	1444 (1001–1888)	750 (750–750)
Accommodation	177 (54–299)	0 (0–0)	169 (44–294)	0 (0–0)	495 (175–815)	0 (0–0)
Food	725 (538–913)	270 (270–400)	719 (527–911)	270 (270–270)	979 (722–1236)	400 (400–400)
Lost income	25 789 (17 488–34 090)	500 (420–26 048)	25 931 (17 438–34 424)	420 (420–25 981)	19 727 (14 129–25 325)	683 (287–27 264)
Postdiagnosis intensive phase						
Direct medical	22 459 (0–47 499)	1086 (0–15 990)	23 976 (0–53 025)	6265 (430–13 626)	13 180 (7325–19 035)	0 (0–16 459)
Travel	4290 (2710–5869)	2588 (0–20 936)	3256 (1760–4751)	0 (0–2960)	33 227 (25 031–41 424)	17 717 (0–44 898)
Accommodation	115 (0–278)	0 (0–0)	0 (0–0)	0 (0–0)	3339 (0–7738)	0 (0–0)
Food	674 (305–1042)	0 (0–428)	440 (97–783)	0 (0–0)	7231 (4553–9910)	0 (0–7858)
Nutritional supplements and extra food	20 665 (15 976–25 353)	20 784 (8660–41 568)	19 310 (14 571–24 048)	16 887 (6928–25 114)	58 588 (42 777–74 399)	34 640 (13 856–69 280)
Lost income	7266 (3031–11 502)	2946 (156–18 692)	6049 (1782–10 315)	721 (52–3268)	41 333 (18 236–64 431)	14 645 (2118–33 074)
Postdiagnosis continuation phase						
Direct medical	7004 (1191–12 817)	466 (0–5456)	6528 (608–12 448)	473 (0–3481)	30 606 (13 979–47 232)	250 (0–12 034)
Travel	5298 (3630–6967)	3138 (226–11 554)	4560 (2946–6174)	1474 (0–4123)	46 776 (28 349–65 203)	17 084 (4805–37 771)
Accommodation	194 (0–443)	0 (0–0)	88 (0–216)	0 (0–0)	6164 (0–18 004)	0 (0–0)
Food	809 (459–1158)	0 (0–721)	706 (358–1054)	0 (0–0)	6552 (3736–9367)	425 (0–6273)
Nutritional supplements and extra food	39 408 (37 458–41 358)	35 506 (35 506–42 434)	39 372 (37 388–41 355)	35 506 (35 506–42 434)	41 434 (37 133–45 735)	35 506 (35 506–44 166)
Lost income	12 715 (1992–23 438)	2583 (403–11 824)	12 095 (1193–22 998)	1271 (206–4695)	47 551 (31 440–63 662)	12 834 (2936–41 821)

Costs are presented in Nepali rupees (NPR). Means, medians, IQRs and 95% CIs are weighted estimates.

### Costs by treatment phase

**Figure 3:**
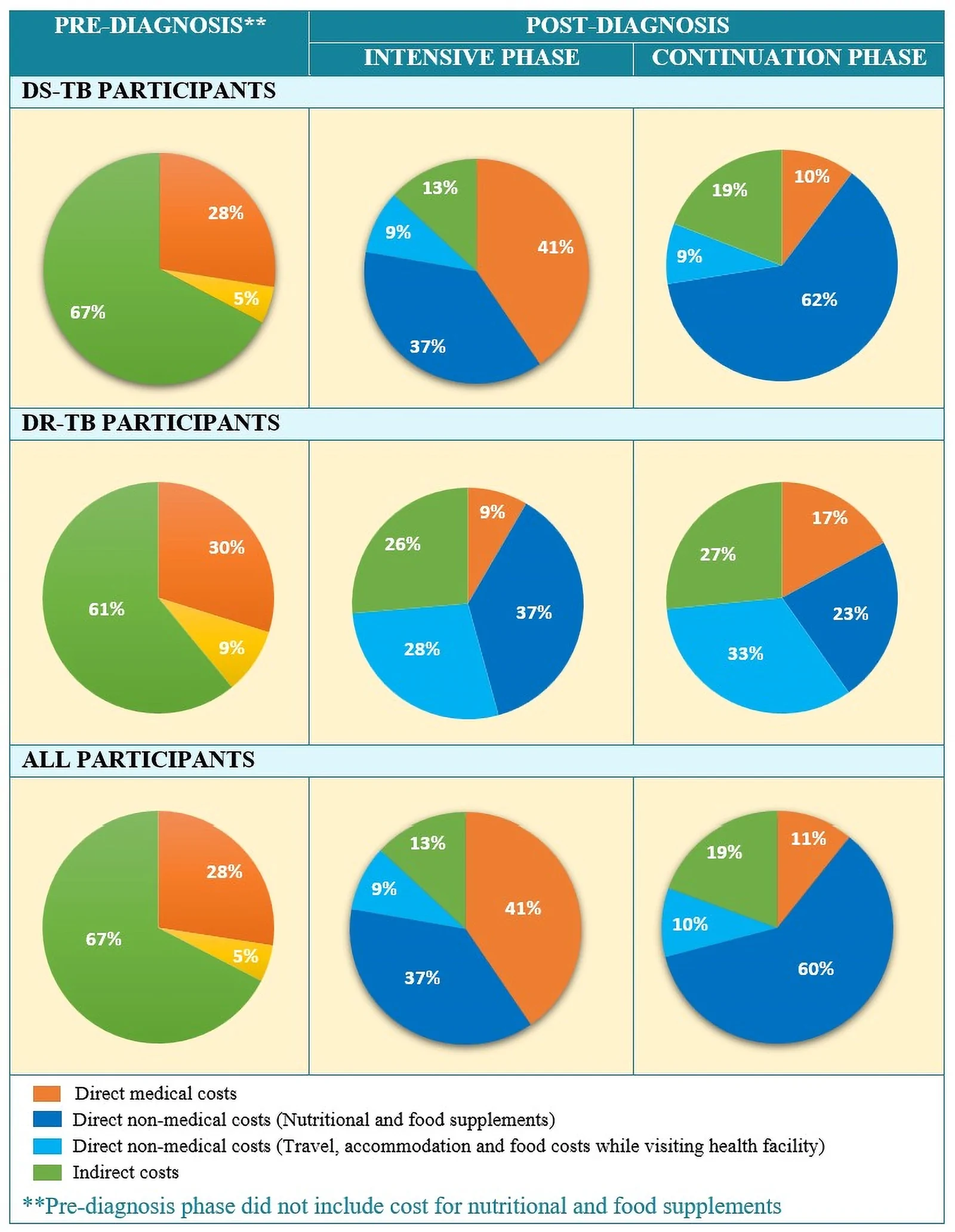
Distribution of costs by treatment phase among TB-affected households in Nepal, 2024 (*N* = 1000).

During the prediagnosis phase, the major proportion (67%) of total costs was the lost income for all participants, with 67% among DS-TB and 61% among DR-TB participants (Fig. 3). Among all participants, direct medical costs were the largest component during the postdiagnosis intensive phase (41%), whereas nutritional supplements and extra food were the largest component during the continuation phase (60%). In the postdiagnosis continuation phase, the direct nonmedical costs with travel, accommodation and food costs while visiting health facilities accounted for the largest proportion (33%) of total costs among DR-TB participants.

The proportion of TB-affected households experiencing catastrophic total costs due to TB was 51% at the 20% threshold of annual pre-TB household income (Table 6). This proportion was substantially higher among DR-TB households than among DS-TB households (76% vs. 51%).

**Table 6:** Proportion of TB-affected households facing catastrophic costs in Nepal, 2024 (*N* = 1000).

Proportion of catastrophic costs	All participants *N* = 1000 Proportion (95% CI)	DS-TB participants *n* = 600 Proportion (95% CI)	DR-TB participants *n* = 400 Proportion (95% CI)
Human capital approach			
Catastrophic	51.1 (45.6–56.5)	50.5 (45.0–56.1)	75.6 (68.0–81.8)
Not catastrophic	48.9 (43.5–54.4)	49.5 (44.0–55.1)	24.4 (18.2–32.0)

Weighted estimates.

### Impoverishment due to tuberculosis-related costs

Before accounting for TB-related costs, 24% of TB-affected households were below the poverty line. This increased to 37% after TB-related costs, corresponding to 13% of households becoming newly impoverished. Among DS-TB households, poverty increased from 24% to 37%, with 13% newly impoverished. Among DR-TB households, poverty increased from 22% before TB-related costs to 53%, with 31% becoming newly impoverished (Supplementary Table S2).

Poverty increased across all wealth quintiles after TB-related costs. The poorest quintile (quintile 5) had the highest poverty headcount both before and after TB-related costs, increasing from 50% to 61%. However, the largest proportion of newly impoverished households was observed in quintile 3, where 18% of households became newly impoverished, followed by quintile 4 at 16% (Supplementary Table S3).

### Coping mechanisms

Overall, 35% of the participants reported borrowing money, with 42% among DR-TB and 31% among DS-TB participants (Table 7). Fewer than 10% had to sell assets (including land, livestock, or gold jewelry) to cover expenses (5.7% of DS-TB patients and 7.7% of DR-TB).

**Table 7:** Coping mechanisms adopted by TB-affected households in Nepal, 2024 (*N* = 1000).

Coping mechanisms	All participants *N* = 1000 (%)	DS-TB participants *n* = 600 (%)	DR-TB participants *n* = 400 (%)
Borrow money			
Yes	344 (35.1)	184 (30.7)	160 (41.8)
No	656 (64.9)	416 (69.3)	240 (58.2)
Sell property			
Yes	64 (6.5)	34 (5.7)	30 (7.7)
No	936 (93.5)	566 (94.3)	370 (92.3)
Coping strategy^a^			
Did not use any strategy	635 (63.5)	405 (67.5)	230 (57.4)
Used one coping strategy	314 (31.4)	172 (28.7)	142 (35.6)
Used two coping strategies	51 (5.1)	23 (3.8)	28 (7.0)

Counts are unweighted; percentages are weighted estimates.

^a^Participants who used either borrowing money or selling property were classified as using one coping strategy, and participants who used both were classified as using two coping strategies.

### Factors associated with catastrophic costs

Compared with households in which the patient was not the primary income earner, households in which the patient was the primary income earner had higher odds of catastrophic costs due to TB [adjusted odds ratio (aOR) 2.81; 95% CI: 1.90–4.14]. Compared with insured households, uninsured households had higher odds of catastrophic costs due to TB (aOR 1.93; 95% CI: 1.15–3.22). Compared with DS-TB households, DR-TB households had approximately three-fold higher odds of catastrophic costs due to TB (aOR 3.06; 95% CI: 1.92–4.87; Table 8).

**Table 8:** Associations of primary income earner status, insurance status, and TB type with catastrophic costs among TB-affected households in Nepal, 2024 (*N* = 1000).

Variable	Unadjusted OR (95% CI)	*P*-value	Adjusted OR (95% CI)	*P*-value	Variables adjusted for
Primary income earner					
Not primary income earner	Ref	–	Ref	–	Gender, age, wealth index, education level, TB type, and comorbidity
Primary income earner	2.84 (2.08–3.89)	<.001	2.81 (1.90–4.14)	<.001	
Insurance					
With insurance	Ref	–	Ref	–	Gender, age, wealth index, education level, primary income earner status, TB type, and comorbidity
Without insurance	1.88 (1.23–2.87)	.004	1.93 (1.15–3.22)	.014	
TB type					
DS-TB	Ref	–	Ref	–	Gender, age, wealth index, education level, primary income earner status, insurance status, and comorbidity
DR-TB	3.03 (2.00–4.59)	<.001	3.06 (1.92–4.87)	<.001	

Adjusted models were specified using a DAG-informed approach, with exposure-specific covariate selection.

Detailed findings from each DAG-informed model are provided in Supplementary Table S4.

## Discussion

This was the first national survey in Nepal to estimate costs faced by TB patients and their households. Although TB care services are provided free of charge in the public sector, 51% of the TB-affected households experienced catastrophic total costs due to TB. This proportion was substantially higher among DR-TB households than among DS-TB households. Direct nonmedical costs were the largest cost component, followed by indirect costs and direct medical costs. Only one-third of patients reported being enrolled in any form of social protection.

By August 2024, 37 countries globally had completed national TB patient cost surveys, including 19 high TB burden countries (World Health Organization (WHO) n.d.). The proportion of households experiencing catastrophic costs has varied widely, from 13% in El Salvador to 92% in the Solomon Islands (Viney et al. 2021, World Health Organization (WHO) n.d.). The pooled average across surveys was 49%, with a substantially higher burden among DR-TB patients (World Health Organization 2024). Nepal’s estimate is therefore close to the global pooled average and similar to findings from several countries, including Lao PDR, Thailand, Vietnam, Indonesia, and the Philippines (Nhung et al. 2018, Chittamany et al. 2020, Ahmad et al. 2022, Florentino et al. 2022, Youngkong et al. 2024).

The median total TB-related cost was NPR 78 397. DR-TB households incurred more than twice the total cost of DS-TB households. Direct nonmedical and indirect costs together accounted for 83% of the total cost burden, indicating that most financial hardship arose outside the formal health system. This pattern is consistent with a previous subnational study in Nepal, in which indirect costs were the main cost driver during both the pretreatment and intensive treatment phases among patients diagnosed through active and passive case finding (Gurung et al. 2019).

The relatively low share of direct medical costs likely reflects the provision of free diagnosis and treatment through the NTP. Patients continued to incur out-of-pocket medical expenses, particularly during the prediagnosis period for consultations and supplementary diagnostic imaging. Additional costs may also arise from hospitalization, management of adverse drug reactions, and ancillary medications for treatment-related side effects. The NTP provides economic support mainly for multidrug-resistant TB patients, including support for hostel accommodation, nutrition and transportation. This economic support amounts to NPR 3000 (∼USD 27) for ambulatory patients and 1000 (∼USD 8) for hostel-based patients per month throughout treatment. DS-TB patients do not routinely receive support, though the government committed to expanding social protection in 2018.

Direct nonmedical costs, especially spending on additional food and nutritional supplements, accounted for a substantial share of total expenditure. Although TB diagnosis and treatment are provided free of charge through the NTP, patients incurred considerable costs for additional food, high-protein foods, and nutritional supplements during treatment. These costs were concentrated in the postdiagnosis period, indicating that the financial burden of TB in Nepal is driven not only by formal medical charges but also by the costs required to sustain patients during prolonged treatment.

TB and undernutrition are closely linked (Bhargava et al. 2013), and nutritional assessment, counseling and support are important components of people-centered TB care (WHO 2025b). However, because this survey did not measure nutritional status or clinical prescription of supplements, reported zero spending on additional food or supplements should be interpreted cautiously. It may indicate absence of additional purchases, but it may also reflect inability to pay, reliance on household food stocks, receipt of informal or in-kind support, or lack of nutritional counseling. Similar findings were reported in Lao PDR and Indonesia, where nonmedical costs particularly spending on nutritional supplements, accounted for a large share of total costs (Chittamany et al. 2020, Ahmad et al. 2022). A consolidated analysis of national patient TB cost surveys also found that direct nonmedical costs represented a substantial proportion of total costs faced by TB patients (Portnoy et al. 2023).

Indirect costs, mainly time loss, accounted for 39% of total costs in this study. This highlights the need for income protection during TB treatment. However, measures such as paid sick leave and job protection may have limited reach in Nepal, where informal employment is common. Targeted cash transfers, wage replacement, and other social protection mechanisms are therefore needed to protect TB-affected households from income loss during treatment.

Overall, the findings suggest a shifting economic burden across the continuum of care. The prediagnosis phase was characterized mainly by out-of-pocket medical expenses related to care-seeking and diagnostic delays, whereas the postdiagnosis period was dominated by nonmedical and indirect costs, particularly nutrition, transport, and time loss. These results suggest that diagnostic reforms, including expanded access to testing, should be accompanied by postdiagnosis social protection that addresses food security and income loss during treatment.

Almost 13% of all TB-affected households were newly pushed below the poverty line after accounting for TB-related costs. The impact was substantially greater among DR-TB households, among whom 31% became newly impoverished. TB-related costs affected households across the socioeconomic distribution. The poorest quintile had the highest poverty headcount both before and after TB-related costs, while the largest proportions of newly impoverished households were observed in quintiles 3 and 4. This suggests that TB-related costs not only deepen poverty among households already living below the poverty line, but also push economically vulnerable households that were previously above the poverty line into poverty. Similar findings have been reported in Benin and Malawi (Kemp et al. 2007, Laokri et al. 2014).

The findings highlight a persistent equity gap in Nepal’s TB response. Even with free clinical services, poorer and economically vulnerable households remain exposed to nonmedical and income-related financial shocks, suggesting that current social protection measures are insufficient to prevent TB-related impoverishment.

In the DAG-informed models, DR-TB, being the primary income earner for the household, lack of insurance coverage and belonging to a poorer wealth quintile were associated with higher odds of catastrophic costs. Similar factors have been reported in studies from Lao PDR, India, and Kenya (Chittamany et al. 2020, Kirubi et al. 2021, Jeyashree et al. 2024). The elevated risk observed among households in which the patient was the primary income earner underscores the “breadwinner effect,” whereby illness-related income loss threatens overall household stability.

These findings support the household-level social protection measures that compensate for income loss and reduce out-of-pocket spending during TB treatment. Although insurance coverage was associated with lower odds of catastrophic costs, this finding should be interpreted cautiously because public and private insurance were combined in the analysis. Further evaluation is needed to assess the effectiveness of Nepal’s national health insurance program in protecting TB-affected households from financial hardship. Such evaluation is particularly important given ongoing implementation challenges, including limited risk pooling, stagnant financing, inadequate local-level empanelment of health facilities, weak institutional capacity, and an outdated benefit package (Ayer et al. 2024).

Although TB treatment and basic diagnostics are provided free of charge by the NTP, our findings demonstrate that biomedical provision alone is insufficient to protect households from financial hardship. Catastrophic costs were driven mainly by direct nonmedical expenditures, particularly nutrition and transport, and by indirect costs from income loss. This indicates that free TB medicines alone cannot eliminate catastrophic total costs. If patients are expected to improve dietary intake during treatment but receive limited nutritional support, these costs are shifted to households, particularly poorer households and those affected by DR-TB.

Social protection programs should therefore explicitly target nonmedical and income-related financial shocks. As highlighted by Fuady et al. (2024), achieving universal social protection requires moving beyond isolated clinical care to provide comprehensive integrated social benefits. An integrated package could combine in-kind nutritional assistance with targeted cash transfers to help address food costs, transport expenditure and income loss. In Nepal, an integrated package could combine nutritional support or food assistance with targeted cash transfers and wage replacement mechanisms, particularly for patients working in the informal sector. Expanding social protection through this multisectoral approach is necessary to mitigate the socioeconomic consequences of TB.

While the NTP provides monthly cash allowances and health insurance coverage for DR-TB patients, the existing social protection framework remains insufficient to eliminate catastrophic costs. Donor-funded assistance is also project-dependent and not consistently available across regions or patient cohorts. Current support services therefore require comprehensive review, stronger structural integration, and more equitable coverage to ensure effective financial protection for all TB-affected households.

### Study limitations

Study limitations should be considered when interpreting these findings. First, the study was conducted in public health facilities and therefore excluded TB patients who did not seek care, received care outside the NTP, or were lost to follow-up. Second, because the study was conducted in public facilities where TB diagnosis and treatment are provided free of charge, costs incurred in the private health sector may not have been fully captured. Third, cross-sectional design may have introduced recall bias, leading to possible overestimation or underestimation of costs, although structured interviews and record checks were used to minimize this risk. Fourth, social desirability bias may have occurred; however, patient confidentiality was maintained during data collection. Finally, this study focused exclusively on costs attributable to the TB episode. Expenditures for the management of comorbid conditions were not collected; therefore, the overall healthcare-related financial burden on households may be higher than reported. This limitation is particularly relevant for the subset of patients with multiple chronic conditions, for whom the 20% catastrophic-cost threshold may be exceeded when all health-related expenditures are considered. However, the TB-specific approach allows direct comparison with the WHO End TB Strategy indicator.

## Conclusion

Our findings demonstrate that the current model of providing free TB diagnosis and treatment in public health facilities in Nepal is insufficient to protect households from severe financial hardship. More than half of TB-affected households experienced catastrophic total costs due to TB, driven predominantly by direct nonmedical expenditures and indirect income loss rather than clinical fees. The economic burden was disproportionately higher among poorer households, primary income earners, individuals with DR-TB, and those without health insurance. TB-related costs pushed a substantial proportion of households, particularly DR-TB–affected households, below the poverty line.

To reduce the economic burden of TB, national policy must move beyond isolated clinical care. This requires a dual approach: accelerating diagnostic pathways to minimize early out-of-pocket medical spending, and integrating the NTP with robust, multisectoral social protection systems. Comprehensive safety nets could include targeted cash support for income and transport costs together with in-kind nutritional assistance. Such integrated support may help reduce TB-related financial hardship and impoverishment in resource-limited settings.

## Supplementary Material

czag095_Supplementary_Data

## Data Availability

The data underlying this article will be shared on reasonable request with the corresponding author.

## Reflexivity statement

The author group reflects diversity in institutional affiliation, disciplinary background, seniority, and geographic location. It includes contributors from government institutions engaged in Nepal’s NTP, national and international research and development organizations, the United Nations system, and independent research organizations and universities. The team includes three women, and most authors are Nepali nationals, with one co-author based outside Nepal. The authors bring expertise in clinical medicine, public health, health economics, program management, epidemiology, research, and policy. Several authors have been directly involved in tuberculosis policy and program implementation in Nepal, providing strong contextual understanding. The authors recognize that this proximity may have influenced the framing, interpretation, and presentation of the study. To address this, the study process involved regular reflection on positionality, assumptions, and potential bias during study design, data collection, analysis, and manuscript development. The diversity of the author group and input from external experts including reviewers helped strengthen the rigor, credibility, and interpretive balance of the study.
