# Supplementary material for: Catastrophic costs and impoverishment among tuberculosis-affected households in Nepal: results from a national patient cost survey
Source: Health Policy Plan. 2026 Jul 24;41(8):1401–15. doi: 10.1093/heapol/czag095 (PMC13586624; doi:10.1093/heapol/czag095)
Supplement: Supplementary file 1 [file czag095_supplementary_data.zip › Supplementary Table 1.docx]

Supplementary Table 1. Distribution of nutritional supplement and extra food expenditure among TB-affected households, Nepal, 2024

| Group | Unweighted  (N) | Unweighted zero expenditure  (n) | Weighted zero expenditure  (%) | Weighted mean,  NPR | Weighted 25th percentile,  NPR | Weighted median, NPR | Weighted 75th percentile,  NPR | Weighted 90th percentile,  NPR | Weighted 95th percentile,  NPR | Maximum,  NPR |
| --- | --- | --- | --- | --- | --- | --- | --- | --- | --- | --- |
| All | 1000 | 60 | 3.1 | 33496 | 22516 | 35506 | 39403 | 50228 | 61486 | 770740 |
| DS-TB | 600 | 18 | 3.0 | 33119 | 22516 | 35506 | 38970 | 49362 | 61486 | 270538 |
| DR-TB | 400 | 42 | 9.1 | 49601 | 34640 | 35506 | 52826 | 88332 | 112580 | 770740 |

NPR: Nepalese Rupee. Note: Percentiles are descriptive weighted percentiles. Zero expenditure indicates no reported expenditure on nutritional supplements or extra food.
