# Supplementary material for: Catastrophic costs and impoverishment among tuberculosis-affected households in Nepal: results from a national patient cost survey
Source: Health Policy Plan. 2026 Jul 24;41(8):1401–15. doi: 10.1093/heapol/czag095 (PMC13586624; doi:10.1093/heapol/czag095)
Supplement: Supplementary file 1 [file czag095_supplementary_data.zip › Supplementary Table 2.docx]

**Supplementary Table 2. Impoverishment before and after TB-related costs, Nepal, 2024**

| **Group** | **Poor before TB costs, % (95% CI)** | **SE** | **Poor after TB costs, % (95% CI)** | **SE** | **Newly impoverished, % (95% CI)** | **SE** |
| --- | --- | --- | --- | --- | --- | --- |
| All | 23.9 (18.6 to 29.3) | 2.70 | 36.9 (30.7 to 43.0) | 3.10 | 12.9 (10.3 to 15.5) | 1.30 |
| DS-TB | 24.0 (18.6 to 29.4) | 2.80 | 36.5 (30.2 to 42.8) | 3.20 | 12.5 (9.8 to 15.2) | 1.40 |
| DR-TB | 21.5 (16.0 to 26.9) | 2.80 | 52.7 (44.0 to 61.4) | 4.40 | 31.2 (24.8 to 37.6) | 3.30 |

SE: Standard Error. Note: Poverty was assessed using the international extreme poverty line of US$3.00 per person per day in 2021 purchasing power parity (PPP). For Nepal, this was converted using the World Bank World Development Indicators (WDI) private-consumption PPP conversion factor for 2021. Post-cost poverty was calculated after subtracting total TB-related costs from annual pre-TB household income. Newly impoverished refers to households that were not poor before TB-related costs but became poor after TB-related costs. Weighted estimates are presented.
