# Supplementary material for: Catastrophic costs and impoverishment among tuberculosis-affected households in Nepal: results from a national patient cost survey
Source: Health Policy Plan. 2026 Jul 24;41(8):1401–15. doi: 10.1093/heapol/czag095 (PMC13586624; doi:10.1093/heapol/czag095)
Supplement: Supplementary file 1 [file czag095_supplementary_data.zip › Supplementary Table 3.docx]

Supplementary Table 3. Impoverishment before and after TB-related costs by wealth quintile, Nepal, 2024

| **Wealth quintile** | **Poor before TB costs, % (95% CI)** | **SE** | **Poor after TB costs, % (95% CI)** | **SE** | **Newly impoverished, % (95% CI)** | **SE** |
| --- | --- | --- | --- | --- | --- | --- |
| Quintile 1, least poor | 8.6 (4.0 to 13.2) | 2.30 | 18.2 (11.7 to 24.7) | 3.30 | 9.6 (4.5 to 14.8) | 2.60 |
| Quintile 2 | 10.8 (5.8 to 15.9) | 2.60 | 21.2 (14.2 to 28.3) | 3.60 | 10.4 (4.7 to 16.1) | 2.90 |
| Quintile 3 | 21.1 (12.6 to 29.6) | 4.30 | 38.8 (27.6 to 50.1) | 5.70 | 17.7 (12.0 to 23.4) | 2.90 |
| Quintile 4 | 35.9 (25.9 to 45.9) | 5.10 | 52.3 (42.8 to 61.8) | 4.80 | 16.4 (10.6 to 22.3) | 3.00 |
| Quintile 5, poorest | 49.9 (39.7 to 60.1) | 5.20 | 60.8 (50.9 to 70.8) | 5.10 | 10.9 (5.2 to 16.7) | 2.90 |

SE: Standard Error. Note: Poverty was assessed using the international extreme poverty line of US$3.00 per person per day in 2021 PPP. For Nepal, this was converted using the World Bank WDI private-consumption PPP conversion factor for 2021. Post-cost poverty was calculated after subtracting total TB-related costs from annual pre-TB household income. Newly impoverished refers to households that were not poor before TB-related costs but became poor after TB-related costs. Weighted estimates are presented.
