# Supplementary material for: Catastrophic costs and impoverishment among tuberculosis-affected households in Nepal: results from a national patient cost survey
Source: Health Policy Plan. 2026 Jul 24;41(8):1401–15. doi: 10.1093/heapol/czag095 (PMC13586624; doi:10.1093/heapol/czag095)
Supplement: Supplementary file 1 [file czag095_supplementary_data.zip › Supplementary Table 4.docx]

Supplementary Table 4. DAG-informed logistic regression models for factors associated with catastrophic costs due to TB among TB-affected households in Nepal, 2024

Model 1. Primary income earner status and catastrophic costs due to TB

| **Variable** | **Unadjusted OR  (95% CI)** | **P-value** | **Adjusted OR  (95% CI)** | **P-value** |
| --- | --- | --- | --- | --- |
| **Primary income earner** |  |  |  |  |
| Not primary income earner | Ref | - | Ref | - |
| Primary income earner | 2.84 (2.08-3.89) | <0.001 | 2.81 (1.90-4.14) | <0.001 |
| **Gender** |  |  |  |  |
| Male | Ref | - | Ref | - |
| Female | 0.70 (0.53-0.94) | 0.019 | 1.17 (0.84-1.64) | 0.341 |
| **Age category** |  |  |  |  |
| 0-14 years | 0.81 (0.40-1.62) | 0.541 | 1.64 (0.74-3.66) | 0.223 |
| 15-24 years | 0.67 (0.44-1.02) | 0.064 | 1.06 (0.63-1.77) | 0.823 |
| 25-49 years | Ref | - | Ref | - |
| 50+ years | 0.83 (0.60-1.15) | 0.26 | 1.17 (0.76-1.81) | 0.458 |
| **Wealth index** |  |  |  |  |
| Quintile 1 (least poor) | Ref | - | Ref | - |
| Quintile 2 | 1.54 (1.04-2.28) | 0.033 | 1.63 (1.05-2.56) | 0.032 |
| Quintile 3 | 2.68 (1.70-4.24) | <0.001 | 2.55 (1.59-4.08) | <0.001 |
| Quintile 4 | 2.81 (1.71-4.62) | <0.001 | 2.72 (1.52-4.84) | <0.001 |
| Quintile 5 (poorest) | 3.65 (2.37-5.63) | <0.001 | 3.35 (1.99-5.63) | <0.001 |
| **Education level** |  |  |  |  |
| No education or illiterate | Ref | - | Ref | - |
| Literate but no formal education | 1.00 (0.58-1.70) | 0.993 | 1.05 (0.62-1.77) | 0.85 |
| Primary/lower secondary school | 1.50 (0.94-2.40) | 0.088 | 1.32 (0.80-2.17) | 0.271 |
| Secondary/higher secondary   and above | 0.69 (0.44-1.10) | 0.117 | 0.98 (0.53-1.80) | 0.937 |
| Not yet started school | 0.62 (0.27-1.41) | 0.252 | 0.58 (0.25-1.37) | 0.209 |
| **TB type** |  |  |  |  |
| DS-TB | Ref | - | Ref | - |
| DR-TB | 3.03 (2.00-4.59) | <0.001 | 2.97 (1.83-4.80) | <0.001 |
| **Comorbidity** |  |  |  |  |
| None | Ref | - | Ref | - |
| One | 1.00 (0.70-1.44) | 0.997 | 1.15 (0.79-1.67) | 0.471 |
| Two or more | 0.70 (0.34-1.47) | 0.341 | 0.89 (0.43-1.82) | 0.739 |

Model 2. Insurance status and catastrophic costs due to TB

| **Variable** | **Unadjusted OR  (95% CI)** | **P-value** | **Adjusted OR  (95% CI)** | **P-value** |
| --- | --- | --- | --- | --- |
| **Insurance** |  |  |  |  |
| Without insurance | 1.88 (1.23-2.87) | 0.004 | 1.93 (1.15-3.22) | 0.014 |
| With insurance | Ref | - | Ref | - |
| **Gender** |  |  |  |  |
| Female | 0.70 (0.53-0.94) | 0.019 | 1.18 (0.84-1.65) | 0.327 |
| Male | Ref | - | Ref | - |
| **Age category** |  |  |  |  |
| 0-14 years | 0.81 (0.40-1.62) | 0.541 | 1.59 (0.70-3.58) | 0.263 |
| 15-24 years | 0.67 (0.44-1.02) | 0.064 | 1.02 (0.61-1.70) | 0.939 |
| 25-49 years | Ref | - | Ref | - |
| 50+ years | 0.83 (0.60-1.15) | 0.26 | 1.22 (0.79-1.89) | 0.358 |
| **Wealth index** |  |  |  |  |
| Quintile 1 (least poor) | Ref | - | Ref | - |
| Quintile 2 | 1.54 (1.04-2.28) | 0.033 | 1.64 (1.05-2.55) | 0.03 |
| Quintile 3 | 2.68 (1.70-4.24) | <0.001 | 2.52 (1.59-3.99) | <0.001 |
| Quintile 4 | 2.81 (1.71-4.62) | <0.001 | 2.65 (1.49-4.72) | 0.001 |
| Quintile 5 (poorest) | 3.65 (2.37-5.63) | <0.001 | 3.21 (1.90-5.42) | <0.001 |
| **Education level** |  |  |  |  |
| No education or illiterate | Ref | - | Ref | - |
| Literate but no formal education | 1.00 (0.58-1.70) | 0.993 | 1.12 (0.66-1.89) | 0.665 |
| Primary/lower secondary school | 1.50 (0.94-2.40) | 0.088 | 1.38 (0.83-2.28) | 0.21 |
| Secondary/higher secondary   and above | 0.69 (0.44-1.10) | 0.117 | 1.06 (0.57-1.95) | 0.859 |
| Not yet started school | 0.62 (0.27-1.41) | 0.252 | 0.58 (0.25-1.34) | 0.198 |
| **Primary income earner** |  |  |  |  |
| Not primary income earner | Ref | - | Ref | - |
| Primary income earner | 2.84 (2.08-3.89) | <0.001 | 2.82 (1.90-4.19) | <0.001 |
| **TB type** |  |  |  |  |
| DR-TB | 3.03 (2.00-4.59) | <0.001 | 3.06 (1.92-4.89) | <0.001 |
| DS-TB | Ref | - | Ref | - |
| **Comorbidity** |  |  |  |  |
| None | Ref | - | Ref | - |
| One | 1.00 (0.70-1.44) | 0.997 | 1.12 (0.77-1.63) | 0.536 |
| Two or more | 0.70 (0.34-1.47) | 0.341 | 0.88 (0.42-1.81) | 0.716 |

Model 3. TB type and catastrophic costs due to TB

| **Variable** | **Unadjusted OR  (95% CI)** | **P-value** | **Adjusted OR  (95% CI)** | **P-value** |
| --- | --- | --- | --- | --- |
| **TB type** |  |  |  |  |
| DS-TB | Ref | - | Ref | - |
| DR-TB | 3.03 (2.00-4.59) | <0.001 | 3.06 (1.92-4.87) | <0.001 |
| **Gender** |  |  |  |  |
| Male | Ref | - | Ref | - |
| Female | 0.70 (0.53-0.94) | 0.019 | 1.18 (0.84-1.65) | 0.327 |
| **Age category** |  |  |  |  |
| 0-14 years | 0.81 (0.40-1.62) | 0.541 | 1.59 (0.70-3.58) | 0.263 |
| 15-24 years | 0.67 (0.44-1.02) | 0.064 | 1.02 (0.61-1.70) | 0.939 |
| 25-49 years | Ref | - | Ref | - |
| 50+ years | 0.83 (0.60-1.15) | 0.26 | 1.22 (0.79-1.89) | 0.358 |
| **Wealth index** |  |  |  |  |
| Quintile 1 (least poor) | Ref | - | Ref | - |
| Quintile 2 | 1.54 (1.04-2.28) | 0.033 | 1.64 (1.05-2.55) | 0.03 |
| Quintile 3 | 2.68 (1.70-4.24) | <0.001 | 2.52 (1.59-3.99) | <0.001 |
| Quintile 4 | 2.81 (1.71-4.62) | <0.001 | 2.65 (1.49-4.72) | 0.001 |
| Quintile 5 (poorest) | 3.65 (2.37-5.63) | <0.001 | 3.21 (1.90-5.42) | <0.001 |
| **Education level** |  |  |  |  |
| No education or illiterate | Ref | - | Ref | - |
| Literate but no formal education | 1.00 (0.58-1.70) | 0.993 | 1.12 (0.66-1.89) | 0.665 |
| Primary/lower secondary school | 1.50 (0.94-2.40) | 0.088 | 1.38 (0.83-2.28) | 0.21 |
| Secondary/higher secondary   and above | 0.69 (0.44-1.10) | 0.117 | 1.06 (0.57-1.95) | 0.859 |
| Not yet started school | 0.62 (0.27-1.41) | 0.252 | 0.58 (0.25-1.34) | 0.198 |
| **Primary income earner** |  |  |  |  |
| Not primary income earner | Ref | - | Ref | - |
| Primary income earner | 2.84 (2.08-3.89) | <0.001 | 2.82 (1.90-4.19) | <0.001 |
| **Insurance** |  |  |  |  |
| With insurance | Ref | - | Ref | - |
| Without insurance | 1.88 (1.23-2.87) | 0.004 | 1.93 (1.15-3.23) | 0.014 |
| **Comorbidity** |  |  |  |  |
| None | Ref | - | Ref | - |
| One | 1.00 (0.70-1.44) | 0.997 | 1.12 (0.77-1.63) | 0.536 |
| Two or more | 0.70 (0.34-1.47) | 0.341 | 0.88 (0.42-1.81) | 0.716 |
